# Isolation and Maintenance-Free Culture of Contractile Myotubes from *Manduca sexta* Embryos

**DOI:** 10.1371/journal.pone.0031598

**Published:** 2012-02-15

**Authors:** Amanda L. Baryshyan, William Woods, Barry A. Trimmer, David L. Kaplan

**Affiliations:** 1 Department of Biomedical Engineering, Tufts University, Medford, Massachusetts, United States of America; 2 Department of Biology, Tufts University, Medford, Massachusetts, United States of America; Université de Technologie de Compiègne, France

## Abstract

Skeletal muscle tissue engineering has the potential to treat tissue loss and degenerative diseases. However, these systems are also applicable for a variety of devices where actuation is needed, such as microelectromechanical systems (MEMS) and robotics. Most current efforts to generate muscle bioactuators are focused on using mammalian cells, which require exacting conditions for survival and function. In contrast, invertebrate cells are more environmentally robust, metabolically adaptable and relatively autonomous. Our hypothesis is that the use of invertebrate muscle cells will obviate many of the limitations encountered when mammalian cells are used for bioactuation. We focus on the tobacco hornworm, *Manduca sexta*, due to its easy availability, large size and well-characterized muscle contractile properties. Using isolated embryonic cells, we have developed culture conditions to grow and characterize contractile *M. sexta* muscles. The insect hormone 20-hydroxyecdysone was used to induce differentiation in the system, resulting in cells that stained positive for myosin, contract spontaneously for the duration of the culture, and do not require media changes over periods of more than a month. These cells proliferate under normal conditions, but the application of juvenile hormone induced further proliferation and inhibited differentiation. Cellular metabolism under normal and low glucose conditions was compared for C2C12 mouse and *M. sexta* myoblast cells. While differentiated C2C12 cells consumed glucose and produced lactate over one week as expected, *M. sexta* muscle did not consume significant glucose, and lactate production exceeded mammalian muscle production on a per cell basis. Contractile properties were evaluated using index of movement analysis, which demonstrated the potential of these cells to perform mechanical work. The ability of cultured *M. sexta* muscle to continuously function at ambient conditions without medium replenishment, combined with the interesting metabolic properties, suggests that this cell source is a promising candidate for further investigation toward bioactuator applications.

## Introduction

Bioactuation involves the use of biological components, such as muscle cells, to generate mechanical force. These types of biological systems could contribute to the miniaturization of actuators for a range of devices, including MEMS and robotic platforms. Several studies have demonstrated the potential for explanted muscles to act as actuators in biotic-abiotic hybrid devices [1], [2]. While these studies serve as valuable proofs of concept, a tissue engineering approach may be more appropriate due to the ability to control dimensions, composition, and force generation of such constructs, in addition to the potential for production scalability. Rat and mouse cardiomyocytes have been the most widely used cell types for tissue engineered bioactuator studies. These cells are attractive for their synchronous and spontaneous contractile abilities, converting chemical energy from glucose into mechanical work [3]. These constructs have mainly been used for basic studies of transport and response times, with the eventual goal of actuating of MEMS devices. For example, rat cardiomyocytes have been cultured on the surface of polydimethylsiloxane spheres and films to perform pumping or bending functions upon contraction [4], [5].

Despite the useful properties of these cells, stringent temperature, pH and osmotic pressure requirements impede the long-term use of such systems unless frequent media changes or a CO_2_-regulated incubator are used. Insect cells are viable candidates to circumvent these problems, as insects survive under a remarkable range of conditions and *in vitro* cultures may be maintained under ambient conditions [6]. Furthermore, insect muscle systems are well-suited as linear actuators since they can have high power output [7], efficiency [8] and strain [9], [10]. For example, in a single work cycle, an isolated 5 mm muscle from the tobacco hornworm, *Manduca sexta* can pull a 3 g weight a distance of 1.4 mm. The ability of these cells to function virtually maintenance-free for a long time highlights their potential as actuators for various devices such as micropumps or soft crawling robots.

The relative simplicity of insect tissues is also important for harnessing muscles for bioactuation applications. Whereas most mammalian muscles contain thousands of fibers, arranged hierarchically, *M. sexta* muscle usually consists of 2–14 single-celled fibers, an arrangement which may be feasible to recapitulate *in vitro*. Furthermore, mammalian muscle relies upon the transport of nutrients and gases through a complex vasculature; in contrast *M. sexta* does not have blood vessels and the transport of oxygen to tissues is mainly by passive diffusion through the tracheal system. Insects are known to adapt to environmental stresses by altering their use of metabolic pathways [11], [12], a feature that may be helpful in future technological applications.

This robustness to conditions is illustrated by a study on dorsal vessel cells (that act as an insect heart) isolated from Lepidopteran larvae that spontaneously contracted at room temperature for 18 days, with half-medium changes every two weeks [13]. This is a promising first step; however, cells isolated from larvae have a developmental history, and therefore may not be as readily manipulated as immature embryonic cells. Although intact *M. sexta* muscle fibers have been isolated from larvae to study their physiology and development, embryonic *M. sexta* myoblasts have not been cultured extensively. We have therefore used isolation methods and cell culture conditions developed for culturing cells from *Drosophila melanogaster* embryos [14] to inform our current approach.

In the present study, our goal was to develop methods to isolate *M. sexta* muscle cells, to generate contractile insect muscle, to characterize the resulting tissue, and to compare metabolic and contractile properties to that of the myogenic mouse cell line C2C12. We have isolated and cultured embryonic *M. sexta* myoblasts, using the developmental hormone 20-hydroxyecdysone (20-HE) to differentiate the cells. Vitellophages or yolk cells were also identified in the culture, and potentially contribute to the extended survival of the muscle in the absence of media replenishment. We were able to suppress differentiation while promoting proliferation using the juvenile hormone mimic, methoprene, which is a significant step toward expanding undifferentiated cells and maintaining them indefinitely as a cell line. When cryopreserved, cells were revived with limited viability, yet retained their capacity for differentiation. Cellular metabolism of the differentiated muscle cells was assessed over time. The *M. sexta* muscle cultured *in vitro* was able to undergo sustained spontaneous contractions without diminishing glucose concentration in the media.

## Materials and Methods

### 2.1. *M. sexta* embryo staging

Eggs were collected from a foam substrate moistened with a tobacco infusion and hung in a flight cage populated by *M. sexta* adults. At the end of each 3 hour collection period, eggs were manually detached from the substrate and maintained at 26°C for an additional 19 hours.

### 2.2. Isolation and culture of *M. sexta* myoblasts

Culture medium was prepared according to Luedeman and Levine [15], with minor modifications. All reagents were purchased from Invitrogen (Carlsbad, CA) or Sigma-Aldrich (St. Louis, MO), unless otherwise indicated. Medium was prepared with 70% Leibovitz's L15 medium, 18% Grace's Insect Medium, 12% fetal bovine serum (FBS), 3.4 mg/mL yeast extract, 3.4 mg/mL lactalbumin hydrolysate (MP Biomedicals, Solon, OH), 0.37 mg/mL α-ketoglutaric acid, 1.21 mg/mL D (+)-glucose monohydrate, 0.67 mg/mL malic acid, 60 µg/mL succinic acid, 60 µg/mL imidazole, 1% Anti-Anti, 0.5% 1× RPMI 1640 vitamin mix, and 0.5% 1× RPMI 1640 amino acid mix. Medium was prepared with 1.14 mg/mL ethylene glycol-bis(2-aminoethyl)-N,N,N′,N′-tetraacetic acid (EGTA) for initial plating and without EGTA for general maintenance and differentiation [14]. Unless otherwise stated, a concentration of 20 ng/mL 20-hydroxyecdysone (20-HE) was used to induce myogenic differentiation. Medium was sterile filtered before use and the pH was adjusted to 6.5 with sterile 1 M NaOH.

After 19 hours of incubation, embryos were counted, washed twice with dH_2_O and sterilized in 25% bleach for 2 minutes. Embryos were then washed twice with water and transferred to a 60-mm Petri dish. EGTA-containing medium was used to wash the embryos once before they were transferred in 5 mL media to a 7 mL Dounce homogenizer (Wheaton, Millville, NJ). Cells were released by lysing the embryos with 6 gentle strokes using plunger B. The homogenate was transferred to a 50 mL conical tube and centrifuged twice, each for 3 minutes at 85×g to remove excessive yolk material and pellet the cells. The pellet was then resuspended in medium and plated at a density of 5 embryos/cm^2^. Plates were incubated at 26°C for 1–2 hours to allow for cell adhesion. Culture dishes were then placed on a rotational shaker for 10 minutes at 100 rpm, 26°C. The cells were gently aspirated and refreshed with an appropriate volume of EGTA-free medium. All plates were sealed with Parafilm and placed in a humidified incubator at 26°C.

### 2.3. LIVE/DEAD staining

Viability and cell death were monitored in cultures maintained long-term without medium refreshment. At designated time points, medium was gently aspirated and the cells were washed once with phosphate buffered saline (PBS). The samples were then stained using the LIVE/DEAD staining kit (Invitrogen). A solution of 4 µM ethidium homodimer-1 and 2 µM calcein-AM in PBS was added to each well and incubated for 30–45 minutes at room temperature. The samples were then washed twice with PBS and imaged using a Leica (Buffalo Grove, IL) microscope with fluorescence capability.

### 2.4. Cell type identification

#### 2.4.1. Histological processing and staining of developing embryos

Embryos were incubated at 26°C for 19 hours as described in Section 2.1. above. The eggs were then pierced with a needle to allow for permeation of the fixative through the egg's outer layer or chorion. The eggs were incubated in 10% neutral buffered formalin for 45 minutes, processed through an ethanol dehydration series into xylene, and embedded in paraffin. Following sectioning, mounting, and deparaffinization,10 µm sections were stained with hematoxylin and eosin. Images were acquired using a Leica inverted microscope equipped with a color camera.

#### 2.4.2. Oil Red O staining

Oil Red O stain was used to view lipid droplets within yolk and/or fat cell types. A 3.5 mg/mL solution of Oil Red O powder in isopropanol was used as the stock solution. Cells were fixed in 4% neutral buffered formalin for 45 minutes. A 60% working solution of Oil Red O stock in PBS wasfiltered through a 0.22 µm filter. Working solution was then added to each well and incubated on a shaker for 45 minutes. The samples were washed thoroughly in PBS and imaged using a Leica inverted microscope equipped with a color camera.

#### 2.4.3. Immunofluorescence staining

Samples for immunostaining were prepared according to Das *et. al.* [16], with minor modifications. Briefly, samples were fixed in cold methanol for 5–7 minutes. Wells were washed with PBS and permeabilized in a solution of 1 wt% bovine albumin serum (BSA) and 0.05 wt% saponin in PBS for 5 minutes. The permeabilization solution was removed before the samples were blocked for 30 minutes in 1 wt% BSA with 10% goat serum. Samples were then incubated overnight at 4°C in primary antibody against waterbug flight muscle myosin (Babraham Institute, Cambridge, UK), diluted 1∶5 in permeabilization solution. The following day, samples were washed twice with PBS and incubated in a solution of AlexaFluor488 F(ab′)2 fragment of goat anti-mouse IgG (1∶200 dilution) and 4′,6-diamidino-2-phenylindole (DAPI, 1∶1000 dilution) for 2 hours. Samples were washed with PBS before visualizing using a Leica fluorescence microscope.

### 2.5. Hormonal dosing

#### 2.5.1. Culture preparation

Media was prepared as described above, except that varying doses of 20-HE or methoprene, a juvenile hormone mimic, were added. Cells were harvested as in Section 2.2, in EGTA-containing media with the appropriate experimental levels of 20-HE or methoprene present.

#### 2.5.2. BrDU incorporation and staining

At each time point, a 0.3 mg/mL BrDU stock was added to each sample for a final media concentration of 3 µg/mL (N = 3). The cells were replaced in the incubator for an additional 3 h, after which the cells were fixed in 10% neutral buffered formalin for 45 min. Cells were permeabilized in 0.1% Triton-X-100 and rinsed with PBS. DNA was denatured for 30 min in 2 N HCl at 37°C. 0.1 M borate buffer (38 mg/mL sodium borate in water, pH 8.5) was used to neutralize the samples. The cells were then washed in PBS and blocked with 10% goat serum. Anti-BrDU mouse monoclonal antibody (Abcam, Cambridge, MA) was diluted 1∶500 in 10% goat serum. Samples were incubated overnight at 4°C. On the next day, samples were washed with PBS prior to incubation in AlexaFluor488 goat anti-mouse secondary antibody (1∶200) and DAPI (1∶1000), diluted with 2% goat serum. After incubation for 1 hour, samples were washed with PBS before imaging with a Leica fluorescence microscope. 7 fields of view were acquired per well, with an average of 55 cells counted per field.

### 2.6. Metabolic comparison with mouse muscle

#### 2.6.1. Culture preparation

Insect cells were plated as described in Section 2.2. After 2 days, the media was aspirated and replaced with fresh control medium (1.23 g/L) or low-glucose medium, which contained 0.27 g/L added glucose monohydrate. At each time point, 35 µL of medium was collected from each well and phase contrast imaging was performed. Medium samples were stored at 4°C until the time of assay.

#### 2.6.2. Culture of cells from established mouse cell line C2C12

P3 C2C12 (CRL-1772 ATCC, Manassas, VA) cells were expanded in T-flasks in growth medium consisting of high glucose Dulbecco's Modified Eagle's medium (4.5 g/L glucose), supplemented with 10% FBS, 1% MEM non-essential amino acids, and 1% Anti-Anti. Cells were detached using a 0.25% trypsin-EDTA solution, centrifuged at 313×*g* for 10 minutes, resuspended and counted. Cells were plated at a seeding density of 5×10^3^ cells/cm^2^. Cultures incubated at 37°C, 20% O_2_, 5% CO_2_. Seven hours following plating, the medium was switched to differentiation medium, which consisted of DMEM supplemented with 10% horse serum, 1% non-essential amino acids, and 1% anti-anti. The cells were then cultured for an additional 2 days before the start of the experiment. At t = 0, the medium was aspirated and replaced with either control differentiation media, or low-glucose media which contained 1 g/L glucose. At each time point, 35 µL of medium was collected from each well and phase contrast imaging was performed. Medium samples were stored at 4°C until the time of assay.

#### 2.6.3. Metabolic assays

Glucose media concentrations were determined using the Glucose (GO) Assay Kit (Sigma). Samples were diluted 40× or 80× and the assay was carried out in 96-well plates according to the manufacturer's protocol. Glucose concentrations were calculated and corrected for losses due to sampling following Equation (1) from Gawlitta *et al.* [17]:
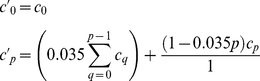
(1)where c_0_ = Initial concentration

c′_0_ = Corrected initial concentration
c_p_ = Concentration of sample p
c′_p_ = Corrected concentration of sample p

Extracellular lactate was evaluated using the EnzyChrom L-Lactate Assay Kit (BioAssay Systems, Hayward, CA) and was performed using 4× or 8× diluted samples in 96-well plates following to the manufacturer's protocol. Cell counts were determined from phase contrast images using ImageJ (NIH) software.

#### 2.6.4. High performance liquid chromatography

Proline and alanine concentrations in media samples were determined using high performance liquid chromatography in gradient elution mode using a Nova-Pak C18 Cartridge (4 µm, 3.9×150 mm, Waters, Milford, MA). The separation module (Alliance 2690, Waters) was equipped with a multi-wavelength fluorescence detector (2475, Waters). An AccQFluor Reagent Kit (Waters) was used for precolumn derivatization of amino acid standards and samples, according to the manufacturer's instructions. Amino acid detection was accomplished at an excitation of 250 nm and emission of 395 nm.

### 2.7. Index of movement analysis

Index of movement analysis was performed according to Fujita *et. al.* [18], with minor modification. Briefly, videos of contracting cells (1600×1200 pixels) were acquired on a Leica microscope with a 20× objective lens using Leica LAS MultiTime imaging software. Five images recorded at 700 ms intervals were analyzed from each video. For a single index of movement value, the first image was subtracted from the remaining four to generate four absolute value images. These were then added together to create a single differential image. The average pixel intensity for the differential image was normalized to a scale of 0 to 1, with 1 as the value representing the greatest displacement in pixel intensity; this value was considered the index of movement. The index of movement reflects the degree of cellular contraction occurring in the field during the 2.8 s interval of data collection. Plots were generated by evaluation the index of movement for at least three videos in three fields of each condition.

### 2.8. Cryopreservation

Cells designated for cryopreservation were isolated as described above in Section 2.2. Following the second centrifugation step, cells were resuspended in growth medium containing either 10% dimethyl sulfoxide (DMSO) or 10% glycerol. Twelve embryos were aliquoted per freezing vial, and stored in liquid nitrogen for at least two weeks. At the start of the experiment, cells were freshly isolated and plated. Simultaneously, frozen vials were thawed, diluted 1∶2 in growth medium +EGTA, and plated. Following 2 hours of incubation at 26°C, all wells were aspirated and replaced with fresh medium (−EGTA).

### 2.9. Statistical analysis

All assays were performed with a minimum sample size of n = 3. Experimental groups were compared using a two-sided Student's t-test in Microsoft Excel. Statistically significant values are defined as indicated.

## Results and Discussion

### 3.1. *M. sexta* cell isolation and culture

Cells were isolated from embryos rather than larvae or pupae, as this approach generated the highest yield of usable cells and avoided the dissection of individual larvae. Additionally, embryonic cells have little developmental history and thus a greater potential for manipulation than lineage developed cells. Our aim was to collect muscle precursor cells, and to induce their differentiation. Therefore, eggs were staged such that myogenesis had begun, but muscle differentiation had not yet occurred. The staging time was determined from the myoblast staging time for *Drosophila*, which is 4 hours [14]. At this point in the Drosophila program of development, gastrulation has begun and germ band extension is underway [19]. These developmental events occur for *M. sexta* around 19 hours post-ovipositioning; thus, a 19-hour staging time was chosen [20]. The staging time is a crucial factor in the generation of myogenic cultures; previous efforts to generate cell lines from *M. sexta* embryos using staging times much further into development, resulted in cultures containing mainly fibroblastic cells [21].

**Figure 1:**
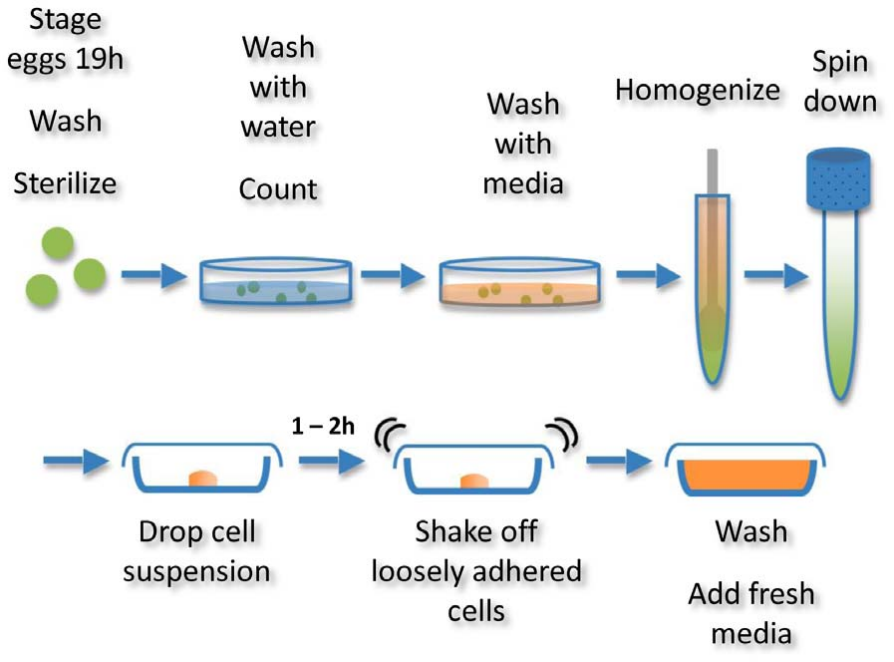
Schematic of embryonic *M. sexta* cell isolation process.

**Figure 2:**
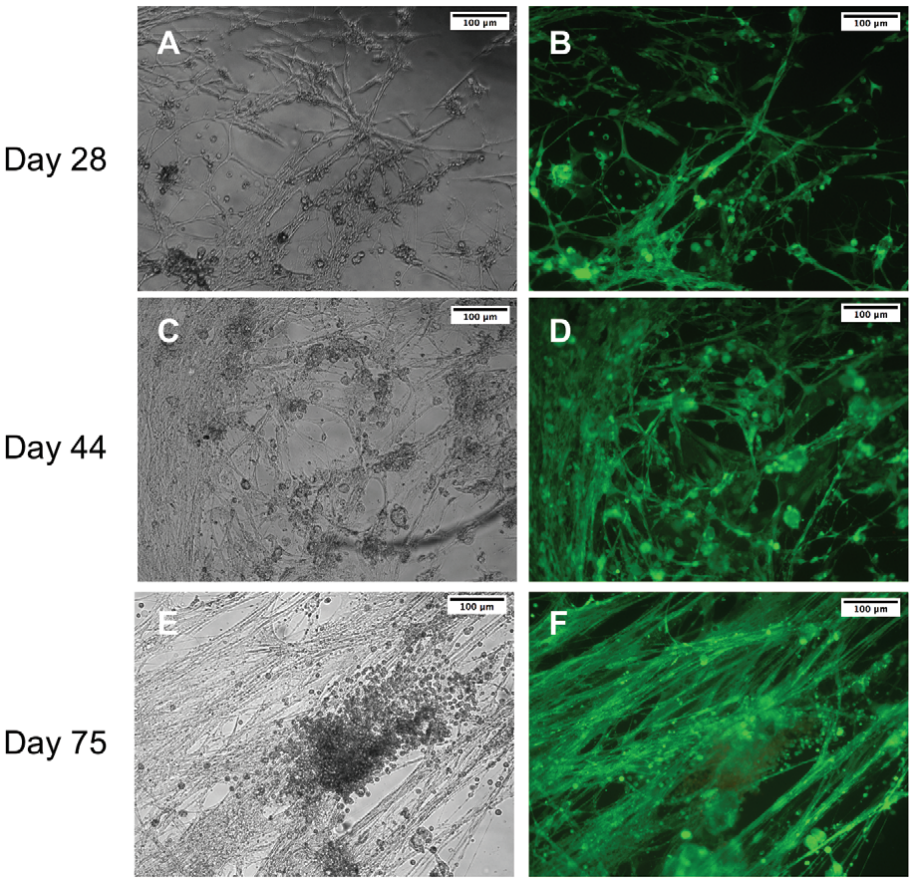
*M. sexta* muscle cell viability over time. Phase contrast (A, C, E) and LIVE/DEAD staining (B, D, E) of typical cultures grown in the absence of medium changes. For LIVE/DEAD images, green staining indicates live cells and red staining indicates dead cells. Representative images taken from cultures on day 28 (A–B), day 44 (C–D), and day 75 (E–F). Scale bars are 100 µm.

**Figure 3:**
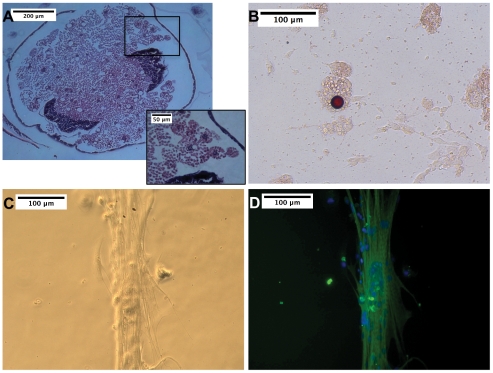
Cell type identification within heterogeneous cultures. Histological sectioning and H&E staining of a developing embryo 19 h post-ovipositioning (A). Dark regions are cross-sections of the embryo. The rest of the egg contains yolk granules and yolk cells. Yolk cells (vitellophages) are 20–50 µm in diameter and contain a single nucleus, stained dark purple (inset, day 0). These cells are also observed in our cultures, and tend to contain a single, large lipid droplet, as shown in red by Oil Red O staining (B). Myogenic cells are the dominant population present in long-term cultures (C–D). Positive staining of myotubes for insect muscle myosin heavy chain (green) on day 48 confirms mature muscle cell identity. Additionally, sarcomeric striations are visible. Nuclear staining with DAPI (blue) reveals multinucleation Scale bars are as noted.

Embryonic cells were isolated using a simple process by which embryos were lysed and the cells were collected by centrifugation (Figure 1). Upon plating, cell masses containing myoblasts adhered to culture dishes. Due to the chelation of free calcium by EGTA, yolk material and most contaminating cell types did not attach; thus, an enriched culture of myogenic cells was generated [14]. Upon plating, myoblasts migrated from tissue masses and readily fused when exposed to low-level doses of 20-HE. Within five days of plating, multinucleated myotubes began to contract spontaneously, possibly due to glutamate presence in the media. This activity persisted throughout the duration of the experiments (>2 months). Cell fusion increased over the course of the culture, resulting in extensive, interconnected myotube networks by day 28, with no evident cell death (Figure 2A–B). These networks continued to develop in the absence of medium changes, and at day 44, cells achieved confluence and nearly all remained viable (Figure 2C–D). By day 75, highly developed bundles of muscle tissue were present and continued to survive and contract, while many of the undifferentiated cells in the culture were no longer viable (Figure 2 E–F). This indicated a metabolic capability unique to the muscle tissue that allowed for extended cell survival under the closed system conditions of the cell culture well, perhaps through breakdown and mobilization of stored nutrients by vitellophages (yolk cells) or trophocytes (fat cells) (Figure 3B). Indeed, researchers have cultured intact embryos *in vitro* and have found them to develop normally [22].

### 3.2. Cell population characterization

Positive staining for insect muscle myosin heavy chain confirmed the myogenic identity of the dominant cell population in the cultures (Figure 3C–D). Sarcomeric striations are visible, however, they are not as precisely aligned as in mammalian muscle (Figure 3D). This is in agreement with the characteristics of larval muscle *in vivo*, where Z-bands are irregular compared to adult and mammalian muscle [23]. Furthermore, mammalian sarcomeres are typically spaced 2 µm apart; however, we observed spacing of 4–13 µm in our cells. Again, this is consistent with physiological studies of larval muscle, where sarcomere lengths are longer than those of mammalian muscle [23]. These ultrastructural differences may contribute to differences we observed in contractile properties, which is discussed in more detail below.

Although our resulting populations are enriched in myogenic cells, several contaminating cell types are present. In particular, cells containing lipid droplets are evident in every culture (Figure 3B). These cells tend to be large, typically 20 to 50 µm in diameter. Although it is possible that some are fat body cells known as trophocytes, which may have differentiated from mesodermal precursors in our cultures, it is more likely that they are yolk cells, or vitellophages, as we tend to observe such cells from the time of initial plating. Vitellophages are preexisting in the eggs at the time of cell isolation and serve to break down the embryonic yolk and may eventually become midgut epithelial cells as well [24], [25]. To verify this hypothesis, we fixed and sectioned eggs developed to the staging time where we normally collect cells. When we stained these eggs with hematoxylin and eosin, we observed large extraembryonic cells containing many small vesicles and a single nucleus (Figure 3A, inset). These cells have a very similar morphology and size to the lipid droplet-containing cells we observe in culture (Figure 3B). It should be noted that the small droplets, while taking up hemotoxylin, do not stain positively for Oil Red O and thus do not contain lipid (Figure 3A–B). Yolk consists of lipids, mainly triacylglycerol, as well as proteins, and a small amount of carbohydrates stored as glycogen [25]. Therefore, the material in the remainder of the vitellophage vesicles is most likely protein or glycogen.

### 3.3. 20-hydroxyecdysone influence on muscle differentiation

During insect development, 20HE acts on multiple cell types and plays important roles in several processes, including molting, metamorphosis, and the coordination of many events during embryogenesis [26], [27], [28]. Its morphogenic functions occur partly through transcriptional upregulation of β tubulin [29]. This is significant for muscle development, since β3 tubulin is transcribed by all somatic myoblasts, and β1 tubulin expression is seen in the apodemes, or muscle attachment sites during embryogenesis [30]. 20HE also acts in the cell cycle to control proliferation through a G_2_ phase control point requiring a suprathreshold level of ecdysteroid [31]. Researchers have described the effects of ecdysteroid dosage on myoblast proliferation and differentiation in *M. sexta* pupae [31]. Although the requirements for dosing may be different for embryonic myoblasts *in vitro*, it is likely that the actions of 20HE may be the same, namely regulating the cell cycle and initiating differentiation.

**Figure 4:**
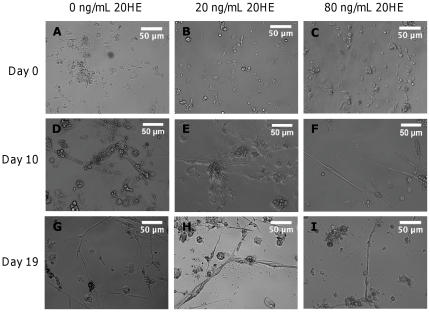
Dosage-dependent effects of 20-HE on muscle differentiation. Phase contrast images showing myotube formation under varying concentrations of 20HE at day 0 (A–C), day 10 (D–F) and day 19 (G–I). Representative images are shown of cells cultured in 0 ng/mL (A, D, G), 20 ng/mL (B, E, H) or 80 ng/mL 20HE (C, F, I). Scale bars are 50 µm.

Our goal was to establish appropriate dosing levels of 20HE for developing larval muscle *in vitro*. Medium concentrations higher than 1,000 ng/mL were observed to be detrimental to cell survival long term (data not shown), so a narrower range of ecdysteroid levels was chosen. Some muscle differentiation did occur in the absence of exogenous 20HE application (Figure 4A, D, G). This indicates that the cells are capable of undergoing myogenic signaling and differentiation to some extent without genetic regulation via 20HE. However, the myotubes formed under this condition were small on day 10 and had deteriorated by day 19 (Figure 4 D, G). When cultured in media containing 20 ng/mL 20HE, myotube formation was evident by day 10, and maturation had continued through day 19, as evidenced by the increased size of the cells (Figure 4B, E, H). At 80 ng/mL 20HE, myotubes form by day 10 but did not mature further (Figure 4C, F, I). Based on these results, 20 ng/mL 20HE was the optimal medium concentration.

In the animal, 20HE is synthesized from cholesterol derived from dietary sterols [25]. Additionally, 20HE may be produced in yolk cells from precursors in yolk granules, and subsequently released to signal gene transcription in embryonic cells starting during the gastrulation phase [32]. It is therefore possible that the muscle formation occurred in the absence of exogenous 20HE due to 20HE production by the cells themselves. Despite this uncertainty, we conclude that low levels of 20HE promote muscle differentiation and contributes to maturation and maintenance of the tissue in larval *M. sexta* cells *in vitro*.

### 3.4. Proliferation using juvenile hormone

Although application of 20HE generated extensive myogenic differentiation, control and timing of differentiation events is important. Specifically, we would like to be able to induce proliferation in the myoblast population prior to differentiation. This would allow for a larger and more uniform initial cell population, and would also bring us closer toward generating a continuous cell line, which could be immortalized and cryopreserved for ease of experimental initiation and culture. To accomplish this, we chose to apply an insect juvenile hormone mimic, methoprene, which in many cases can modify the effects of 20HE; specifically, it has the ability to suppress 20HE-induced morphogenesis without interfering with 20HE-induced proliferation [31].

**Figure 5:**
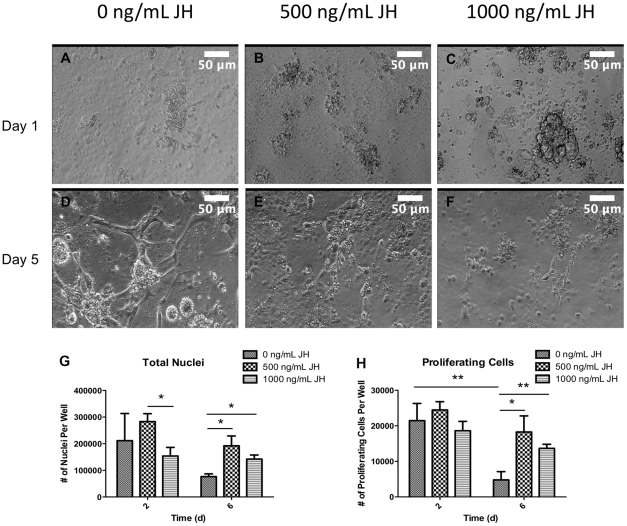
Effects of juvenile hormone (JH) mimic, methoprene, on 20HE action and cell proliferation. Phase contrast images of cells cultured in 20 ng/mL 20HE and varying levels of methoprene (A–F). Total number of nuclei and BrDU-positive nuclei on day 2 and 6 for varying methoprene concentrations (G–H). * p<0.05, ** p<0.01. Scale bars are 50 µm.

On day 1, all cultures contained undifferentiated cells but by day 5, only cells cultured in the absence of methoprene underwent myogenic differentiation (Figure 5A–F). This result was further confirmed by cell counts and BrDU incorporation and staining. The total number of cells were significantly higher on day 6 in cultures exposed to methoprene than cultures with no methoprene (Figure 5G). Cell counts were significantly higher for 500 ng/mL JH compared to 1000 ng/mL JH samples on day 2 but not day 6 (Figure 5G). There were significantly fewer proliferating cells on day 6 than day 2 for control samples; therefore methoprene exposure extends and maintains the proliferative phase in these cells (Figure 5H). Some studies suggest that juvenile hormone action in promoting proliferation is steroid independent [33], so this may be a direction to pursue in future studies.

### 3.5. Metabolic evaluation

**Figure 6:**
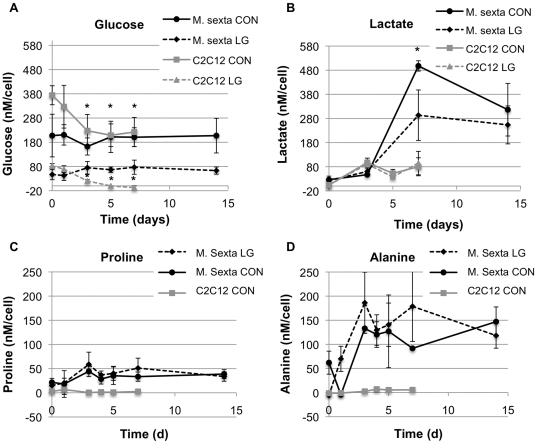
Medium concentrations of metabolites tracked over time in insect and mouse C2C12 cell cultures, on a per cell basis. Glucose consumption and lactate production were analyzed for *M. sexta* control (CON, black circles) and low glucose (LG, black diamonds) conditions, along with C2C12 CON (gray squares) and LG (gray triangles) samples for comparison (A–B). Statistically different values are compared with corresponding time 0 values for glucose (A), and between *M. sexta* CON and LG for lactate (B). For amino acid analysis, C2C12 low glucose condition was omitted (C–D). * p<0.05.

One of our hypotheses was that caterpillar muscle, given its ability to function without a closed circulatory system and with transport of oxygen arising mainly from passive diffusion, could function under nutrient-deprived conditions. Vascularization is a major limiting factor in engineering dense tissues such as muscle [34]. Therefore, if this obstacle could be bypassed it would be a major advantage for the development of bioactuators from insect muscles. Metabolic profiles of *M. sexta* and mouse C2C12 myotubes were evaluated under normal (1.23 g/L and 4.5 g/L, respectively) and low glucose (0.27 g/L and 1 g/L, respectively) conditions. Glucose consumption, lactate production, and cell numbers were quantitatively tracked over time (Figure 6A–B).

C2C12 mouse control and low glucose conditions resulted in steady glucose consumption and lactate production over the first three days, as expected. After this time, activity plateaus, presumably due to nutrient limitations leading to senescence and cell death (Figure 6A–B). Surprisingly, insect control and low glucose cultures do not appear to consume glucose throughout the duration of the experiment. However, after three days, a dramatic increase in lactate production occurs (Figure 6A–B). Despite the high levels of lactate produced, cells remain viable and contractile. Insect cells are able to tolerate a wide range of pH environments, and therefore acidosis was not an issue. The approach utilized by these cells to tolerate pH lowering is unknown, however a mechanism for maintaining intracellular pH, such as a H+-V-ATPase in the plasma membrane, may be at play [35].

The data suggest that, while mouse cells rapidly consume glucose and produce lactate, insect cells have different metabolic activity on a per cell basis. The control group of C2C12 cells consumed nearly 40% of their initial glucose within 3 days and low glucose cultures depleted all of their glucose supply within 5 days. Insect cells, however, did not consume significant amounts of glucose throughout the duration of the culture, which was extended to 14 days for *M. sexta* groups. It is possible that glucose consumption was affected by the action of 20HE, as the hormone may block glycolysis [36]. Another possibility is that glycogen stores in yolk and/or fat cells may have been mobilized to provide a continuous level of glucose in the system. Mouse cells rapidly produced lactate when cultured for several days without medium changes, which was not surprising. Lactate is produced under anaerobic conditions, when cells are starved for a crucial metabolite such as glucose and are forced to convert pyruvate to lactate. The insect cells, after a 3-day lag phase, produced lactate at a rapid rate.

The fact that a large amount of lactate was produced in the absence of glucose consumption led us to consider what energy source the cells were using. Insects have the ability to utilize carbohydrates, amino acids, lipids, or a combination of these for changes in energy demand during flight [37], [38], [39]. Therefore, we investigated whether proline metabolism was contributing to lactate production in the cells. Amino acid profiles were obtained from HPLC analysis of cell culture media, and normalized to amino acid levels in the starting media. C2C12 low glucose samples were omitted from this analysis. For C2C12 control cells, proline was consumed by day 3, at which time a slight increase in alanine concentration was observed (Figure 6C–D). For both insect control and low glucose conditions, alanine levels increased dramatically with respect to starting medium concentrations and proline levels did not significantly decrease (Figure 6C–D). This response is similar to the steady increase in lactate concentration while the levels of glucose remained constant (Figure 6A–B). These data suggest that there may be a mechanism in the cultured cells for producing energy sources such as glucose and proline, allowing their concentrations in the medium to remain stable while generating metabolites. Yolk cells derived from the developing eggs may be responsible for such processes, as their role in development is to break down yolk into a useable form for the embryo.

A third potential candidate for energy metabolism is the lipid stored in the vitellophages. Triglyceride breakdown to diacylglycerol (DG) occurs in the fat body of adult *M. sexta* and other insects, and subsequent transport of DG to muscle tissues provides substrates for free fatty acid metabolism and energy production [40], [41].

### 3.6. Index of movement analysis

Qualitatively in culture, we observed that the frequency and displacement of cells undergoing spontaneous contractile activity varied dramatically between mouse C2C12 cells and insect *in vitro* developed muscle. C2C12 cells tend to have twitch-like contractions that are rapid, with a duration on the order of milliseconds, and have a regular frequency, with one or a region of cells pulsing synchronously for a short period of time, then shutting down permanently. *M. sexta* muscle cells, however, tend to contract with irregularity and more slowly than mammalian muscle, with the time scale for a single contraction duration on the order of hundreds of milliseconds. Additionally, contractions persist throughout the entire duration of the culture.

**Figure 7:**
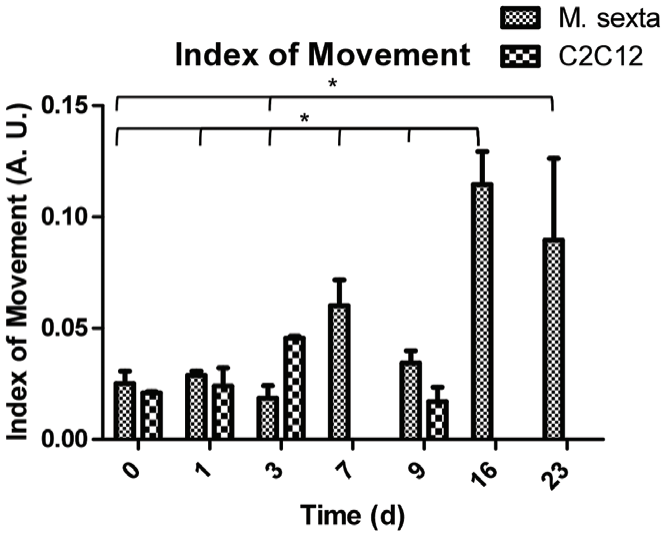
Index of movement analysis for *M. sexta* vs. C2C12 muscle. Plot of average index of movement over time for both cell types for 3 regions per condition. Values are significant for *M. sexta* t = 16d compared to previous time points, as indicated, and t = 23d compared to previous time points, as indicated. * p<0.001.

The spontaneous contractile activity of the cells was analyzed by evaluating the index of movement (IOM) for cultures over time. IOM is a measure of the changes in pixel intensity, and thereby the movements of cells, in a sequential set of frames taken from a video of contracting cells. We subtracted frames 700 ms apart from an initial, baseline image to give differential maps showing the regions that differ in pixel intensity from the reference. We then summed these differential images to provide a snapshot of the cells' activity for a 2.8 s period. The average pixel values of these differential images is the IOM and can be used as a measure of how much the cells moved. We analyzed IOM for each condition at each time point to compare contractile activity of insect MSMY and mouse C2C12 cells over time without medium changes. Confluent C2C12 cells tended to only display contractions in single cells or small areas of the well. However for the insect cells, myocytes throughout the field contracted and, with the exception of a few non-myocytes, nearly the entire field was displaced. Videos were taken from at least 3 regions in insect and mouse samples to track IOM with time (Figure 7). On the third day after contractions began, mouse muscle experienced a peak in movement intensity, followed by contractile arrest. Insect muscle cultures experienced a contractile peak on day 16, and continued to contract at all time points.

One factor that plays a role in the enhanced contractile properties of *M. sexta* muscle is its limited adherence to tissue culture plastic. While C2C12 cells tend to attach to the cell culture substrate along their entire length, insect muscles tend to span from one cell mass to another, allowing them to be suspended with attachments to the cell masses at either end. The distribution of integrins along the cell membrane may play a role in this phenomenon, as well as signaling occurring within the cell masses, which the muscle cells extend from. The mechanical properties of the cells themselves also contribute to their contractile behavior. *In vitro* mouse cells often tear themselves apart during contractile activity, presumably due to their adhesion to the tissue culture plastic and inability to undergo large deformations. However, insect muscle has the ability to stretch more than 160% its resting length, and has been compared to particle-reinforced rubber in terms of its material properties [9], [10]. Muscle contractions occur through changes in sarcomere length, with thin and thick filaments sliding over one another and thus creating shortening in myofibrils. The fact that insect larval muscle has less organized and often longer sarcomeres may allow it more freedom in range of shortening and may favorably influence the material properties. Indeed, sarcomeric striations in *M. sexta* cultures consist of domains space between 4 and 13 µm, rather than the 2 µm spacing typically observed in mammalian striated muscle (data not shown). Contractile properties may also be affected by active zone distribution. Mammalian muscle has a single region of concentrated active zones per cell, while insects tend to have more widely distributed active zones.

In a bioactuated system, we would like to be able to operate for extended periods of time without the need for tissue repair or replacement. Additionally, the force generated by the contractions must persist long enough to perform locomotion or pumping work. Therefore, since insect muscle cell contractions persist over the course of months, and a single contraction produces force for seconds rather than milliseconds, they are well-suited for bioactuation applications. However, in future work, we will develop electrical stimulation regimes such that spontaneous activity may be precisely controlled.

### 3.7. Cryopreservation and viability long-term

**Figure 8:**
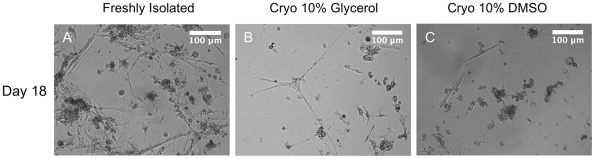
Cryopreservation and resuscitation of *M. sexta* embryonic cells. Phase contrast images of freshly isolated (A) and resuscitated cells, after two weeks of cryopreservation, using DMSO and glycerol as cryoprotectants (B–C)). All panels show day 18 results. Scale bars are 100 µm.

In order to maintain a consistent cell population with a readily available supply of cells, the cryopreservation of the *M. sexta* cells was addressed. Glycerol and DMSO were tested as cryoprotectants, and storage times of at least two weeks were used. Upon resuscitation, centrifugation to remove cryoprotectants from the media resulted in cell lysis, since no cells were observed after plating (data not shown). Therefore, the protocol was adjusted so that frozen cells were diluted 1∶2 in normal media and plated directly. After an hour of incubation to allow for cell attachment, the cryoprotectant-containing medium was replaced with fresh media. Freshly isolated cells grew, differentiated and contracted spontaneously as expected (Figure 8A–B). We found that fewer cells survive cryopreservation in 10% DMSO media than 10% glycerol, however cells obtained from both conditions differentiated into contractile myotubes (Figure 8C–F).

### 3.8. Application as bioactuator device

As the fields of robotics and MEMS develop, the need for an actuator platform on a meso scale has emerged. The advantages of using muscles as actuators include flexibility, silence, biomimetic action, along with the potential for facile production scale-up. Most interesting perhaps, are the potential for such constructs and devices to self-assemble, regenerate or heal, and biodegrade, properties that yet to be imagined in current robotic systems. Though issues of sterility, temperature sensitivity, and real-world robustness have yet to be addressed, we believe that robotic and microsystem actuation may be achieved via bioactuation using the muscle cell system we have presented. Future work will focus on elucidating the metabolic mechanisms of cell survival, and generating 3-dimensional muscle tissue constructs that may serve as linear actuators.

We have demonstrated that embryonic myoblasts from the insect *M. sexta* may be differentiated into functional muscle through the application of the molting hormone, 20-hydroxyecdysone. The resulting multinucleated cells express myosin heavy chain, and a subpopulation of cells exists, which contain lipid vesicles. Metabolic evaluations revealed that these cells do not appear to consume glucose, but they produce more lactate than mouse muscle on a per cell basis. This indicates that the cells use an alternate energy source, such as glycogen reserves, amino acids, or lipids, to fuel cellular activity and spontaneous contractions. As a result, *M. sexta* muscle cells are able to thrive for extended periods of time without medium changes. We have demonstrated that the contractile ability of these cells exceeds that of conventional mammalian systems. Taken together, the larval muscle tissue we have generated from embryonic cells is a promising candidate for future bioactuation applications.
